# ﻿Four new species of *Drymonia* (Gesneriaceae) from South America: Tributes to inspirational leaders

**DOI:** 10.3897/phytokeys.256.148263

**Published:** 2025-05-12

**Authors:** John L. Clark

**Affiliations:** 1 Marie Selby Botanical Gardens, 1534 Mound St., Sarasota, FL 34236, USA Marie Selby Botanical Gardens Sarasota United States of America

**Keywords:** Andes, biodiversity, Colombia, Ecuador, nomadic climber, Peru, taxonomy

## Abstract

Exploratory field expeditions and herbarium research have led to the discovery of four new species of *Drymonia* (Gesneriaceae), distinguished by laterally compressed corollas, elongate inflorescence axes, and a nomadic climbing habit. These species, found in Andean forests of Colombia, Ecuador, and Peru, are unique within the Gesneriaceae for bearing inflorescences on a leafless portion of the stem near the ground, while their foliage is restricted to the subcanopy. Named in honor of visionary and inspirational leaders dedicated to advancing conservation, science, and education, the new species are *Drymoniaclavijoiae* J.L.Clark, **sp. nov.**, *D.katzensteiniae* J.L.Clark, **sp. nov.**, *D.rominieckiae* J.L.Clark, **sp. nov.**, and *D.silvaniae* J.L.Clark, **sp. nov.**

## Abstract (es)

Expediciones de campo y revisiones de herbario han llevado al descubrimiento de cuatro especies nuevas del género *Drymonia* (Gesneriaceae), las cuales se distinguen por sus corolas lateralmente comprimidas, ejes de inflorescencia alargados y hábito de bejuco con crecimiento libre. Estas especies de los bosques andinos de Colombia, Ecuador, y Perú, son únicas dentro de Gesneriaceae por desarrollar inflorescencias en segmentos afilos del tallo, localizadas cerca del suelo, mientras que su follaje está restringido al estrato del subdosel. Nombradas en honor a líderes visionarios e inspiradores dedicados a la conservación, la ciencia y la ed­ucación, las nuevas especies son *Drymoniaclavijoiae* J.L.Clark, **sp. nov.**, *D.katzensteiniae* J.L.Clark, **sp. nov.**, *D.rominieckiae* J.L.Clark, **sp. nov.**, and *D.silvaniae* J.L.Clark, **sp. nov.**

## ﻿Introduction

*Drymonia* Mart. is the third-largest genus within the Neotropical Gesneriaceae, surpassed only by *Columnea* (210+ species) and *Besleria* (175+ species) (Clark et al. 2020; GRC 2025). The genus includes 91 accepted species (including the four described here), with the majority concentrated in lowland, premontane, or montane evergreen wet forests in the northern Andes and the Chocó biogeographic region, particularly in Colombia (44 species) and Ecuador (42 species). Phylogenetic analyses based on molecular data strongly support the monophyly of *Drymonia* (Clark et al. 2015; Ogutcen et al. 2021). Taxonomically, *Drymonia* belongs to the subtribe Columneinae, which accounts for approximately 16% (about 525 species) of the overall species diversity within Gesneriaceae (Weber et al. 2013, 2020).

*Drymonia* is a highly variable genus characterized by significant diversity in leaf morphology, indumentum, corolla shapes and coloration, and fruit types. The habit ranges from herbs, shrubs, small trees, herbaceous vines, and woody lianas. The scandent growth habit is best described as a nomadic climber (Moffett 2000), where plants germinate terrestrially, climb other vegetation via scandent stems or adventitious roots, and may shed older stem portions as they ascend (Zotz 2013).

Corolla forms in *Drymonia* range from campanulate, tubular, infundibuliform, and hypocyrtoid (with a ventral pouch and apical constriction) and display a wide range of colors. The corolla lobe margins vary from entire and crenate to highly dissected, such as laciniate or fimbriate. Anthers in the genus predominantly dehisce through basal pores, historically considered diagnostic for *Drymonia* (Wiehler 1983). However, two phylogenetic studies based on molecular sequence data (Clark et al. 2006, 2015) revealed that at least two clades within the genus exhibit longitudinal dehiscence, a trait derived from poricidal dehiscence, supporting the convergence of longitudinal dehiscence or independent loss of poricidal anther dehiscence within *Drymonia*.

Fruit morphology in *Drymonia* falls into the following four main categories (Clark and Clavijo 2022): (1) fleshy display capsules lacking a distinct endocarp are the most common fruit type found in *Drymonia* as exemplified in the four species described here; (2) fleshy capsules with tardily dehiscent endocarps; (3) fleshy capsules with indehiscent endocarps; and (4) berries.

The inflorescences for most *Drymonia* are characterized as axillary and clustered on short peduncles. Technically, all inflorescences of Gesneriaceae are variations of pair-flowered cymes (Weber 1978, 1982, 1995, 2013). The inflorescence axes in the species described here are unusual because they are pendent and grow zig-zag, often reaching 30+ cm in length and occasionally exceeding 45 cm. These inflorescences were characterized by Weber (1982, 1995) as pair-flowered cymes that are elongated via displaced bracteoles, but the developmental information for studying bracteole displacement (e.g., heterochrony) is not currently available (Weber 1995).

Ongoing studies on the genus *Drymonia*, which involve extensive fieldwork and herbarium research, have revealed four new species characterized by laterally compressed corollas, a nomadic climbing habit, and elongated, zig-zagging inflorescences. These four new species, found on the eastern slopes of the northern Andes, have often been misidentified as *Drymoniacoccinea* (Aubl.) Wiehler. However, they are highly divergent and geographically disjunct from *D.coccinea*, which is restricted to evergreen wet forests in lowland Amazon and the Guiana Shield.

Based on molecular sequence data (Clark et al. 2015), two of the four newly described species are strongly supported as sharing a recent common ancestor. The clade containing these two species is defined by the presence of laterally compressed corolla tubes, a synapomorphy shared with the closely related species *D.hoppii* (Mansf.) Wiehler, *D.affinis* (Mansf.) Wiehler, *D.pendula* (Poepp.) Wiehler, and *D.doratostyla* (Leeuwenb.) Wiehler (see phylogenetic hypothesis in fig. 3 of Clark et al. 2015).

One of the challenges of studying the taxonomy and systematics of the four species described here is that the reproductive structures are typically located on a leafless portion of the stem near the ground, while the foliage tends to be located in the subcanopy (approximately 10 meters above the ground). Flowers are frequently photographed and collected, but foliage is typically absent from herbarium specimens or limited to a single leaf. This is because the process of tearing down or removing the elongated lianas from the tree frequently results in the loss of most leaves when preparing herbarium specimens.

## ﻿Taxonomic treatment

### ﻿ Drymonia clavijoiae

Taxon classificationPlantaeLamialesGesneriaceae

J.L.Clark sp. nov.

A5DFA55D-CB29-5872-97D7-4EA7A4C114D2

urn:lsid:ipni.org:names:77361651-1

Figs 1 , 2

#### Diagnosis.

Differs from all other congeners by the presence of a conspicuous gelatinous residue often covering the inflorescence and flowers. The elongated tubular and laterally compressed flowers in *Drymoniaclavijoiae* are similar to those in *D.coccinea*, but *D.clavijoiae* is also distinguished by an elongated inflorescence axis that often exceed 20 cm in length (versus consistently less than 10 cm in *D.coccinea*). Additionally, the inflorescences in *D.coccinea* lack a gelatinous residue, which is a defining feature of *D.clavijoiae*.

#### Type.

Ecuador • Zamora-Chinchipe: parroquia Los Encuentros, Estación Experimental El Padmi (Universidad de Loja), northern outskirts of El Padmi, 5 km from the town of Los Encuentros, 3°43'S, 78°39'W, 850–915 m, 3 June 2007, *J.L. Clark & Gesneriad Research Expedition Participants 9943* (holotype: US-2 sheets! [barcodes US00961563 & US00961562]; isotypes: AAU, COL, MO, NY, QCNE, SEL! [barcode SEL065837]).

#### Description.

Elongate scandent subwoody nomadic lianas with leaves in the subcanopy (ca. 10 m above ground) and flowers produced near the forest floor along a leafless portion of the stem. ***Stems*** elongate and subwoody, terete in cross section, 4–9 mm in diameter. ***Leaves*** opposite, equal in a pair; petiole 1–5 cm long, green, terete in cross-section; blade oblong to ovate, 8–22 × 2–9 cm, coriaceous, adaxially and abaxially green, apex acute to acuminate, base acute, margin entire, 5–7 pairs of secondary veins, sparsely pubescent with single-celled trichomes abaxially, adaxially glabrous. ***Inflorescences*** covered in a gelatinous residue, produced along a leafless region of stem near ground, of a pair-flowered cyme that elongates from displaced bracteoles, often reaching 30 cm in length, each inflorescence branch subtended by a pair of persistent bracts; each bract uniformly puberulent, oval and red, ca. 1.5 × 1.5 cm; inflorescence with one mature flower open at a time. ***Flowers*** tubular and laterally compressed; pedicels 5–8 mm long. Calyx white to white suffused with red, uniformly puberulent on outside and glabrous on inside, lobes 5, nearly free, fused at the base for 2–4 mm, overlapping, imbricate, and clasping corolla tube, each broadly ovate, apex rounded, base broadly ovate, margins entire, ventral and lower lobes ca. 2.5 × 1.8 cm, the dorsal lobe slightly smaller, ca. 1.4 × 1.3 cm. Corolla tube zygomorphic, protandrous, oblique relative to calyx, 4–5 cm long, gibbous at base, constricted laterally throughout, 1.0–1.3 cm wide, outside uniformly puberulent, inside mostly glabrous with minute glandular trichomes in the upper region of the throat, throat elliptic in cross section and nearly constricted laterally, lobes 5, subequal, margins entire to serrulate, lobes reflexed, 8–11 × 9–12 mm, upper lobes always yellow, lower and lateral lobes yellow with brown spots or uniformly yellow. ***Androecium*** of 4 didynamous stamens, included, filaments broad and flat, ca. 3.5 cm long, adnate to the corolla tube base for 4 mm, white, glabrous; anthers oblong, sagittate, coherent by the lateral walls, initially dehiscent by basal pores that later develop into longitudinal slits, 4.2–6.0 × 0.7–2.0 mm. ***Gynoecium*** with a single bilobed dorsal gland; ovary superior, 4.0–5.0 × 4.0–5.0 mm, cone-shaped, puberulent; style stout, included, 3.2 cm long; stigma stomatomorphic. ***Fruit*** a bivalved fleshy capsule, valves light red and reflexed when mature, each valve 1.3 × 1.3 cm. **Seeds** numerous, 0.8–10.0 × 0.4–0.5 mm, light brown, fusiform, ridged.

#### Additional specimens examined.

**Colombia.** • **Cauca**: cantón Santa Rosa, carretera Macoa-Pitalito, 928 m, 1°20'25.45"N, 76°32'12.51"W, 2 Aug 2024, *J.L. Clark et al. 19102* (COL, HEEA, SEL). **Ecuador.** • **Morona-Santiago**: cantón Limón Indanza, Cordillera del Condor, trail towards crest of the Cordillera del Condor from camp #1 (10–15 km S/SE from the comunidad Warints), 830–1200 m, 3°13'S, 78°15'W, 17 Dec 2002, *J.L. Clark 7058* (QCNE, US); • cantón San Juan Bosco, road between San Juan Bosco and El Pangui, 27 km south of San Juan Bosco, 1591 m, 3°17'51"S, 78°33'27"W, 2 Jun 2007, *J.L. Clark et al. 9904* (ECUAMZ, US); • road Limón to Mendez, Aug 1989, *A. Hirtz & X. Hirtz 4413* (SEL); • Cordillera de Cutucú, western slopes, along a trail from Lograño to Yaupi, 700 m, Nov 1976, *M.T. Madison et al. 3151 et al.* (SEL, US); • Macas, along new road, west into the Andes, first 17 km westward, then ca. 12 km south on side road, 13 Apr 1988, *H. Wiehler et al. 8805* (SEL, US); • Cordillera de Cutucú, between Sucua and Patuca, 17 Apr 1988, *H. Wiehler et al. 8858* (SEL). • **Napo**: cantón Archidona, Reserva Ecológica Antisana, comunidad Shamato, entrada por km 21 – Shamato, camino Sardinas – Shamato, 1700 m, 0°44'S, 77°48'W, 27 Apr 1998, *J.L. Clark, E. Narváez & G. Alvarado 5232* (QCNE, US); • cantón Archidona, parroquia Catundo, buffer zone of Parque Nacional Sumaco Napo Galeras, comunidad Mushullakta, 900–970 m, 0°47'49"S, 77°35'10"W, 24 Feb 2003, *J.L. Clark, N. Harris & L. Narváez 7206* (F, K, MO, QCA, QCNE, SEL, US); • Tiputini Biodiversity Station (Universidad San Francisco, Quito), Yasuní Biosphere Reserve, near Río Tiputini, 200 m, 0°38'11"S, 76°8'58"W, 24 Jun 2006, *J.L. Clark, L. Bohs & I. Nenquimo 9473* (ECUAMZ, MO, NY, SEL, US); • Yasuní Biosphere Reserve, Tiputini Biodiversity Station (Universidad San Francisco, Quito), sendero Chichico to sendero Parahuaco, 200 m, 0°38'11"S, 76°8'58"W, 17 May 2007, *J.L. Clark et al. 9571* (ECUAMZ, MO, NY, SEL, US); • Yasuní Biosphere Reserve, Tiputini Biodiversity Station (Universidad San Francisco, Quito), sendero Maquisapa to sendero Harpía, 200–250 m, 0°38'11"S, 76°8'58"W, 22 May 2008, *J.L. Clark et al. 10233* (ECUAMZ, SEL, US); • cantón Archidona, parroquia Catundo, buffer zone of Parque Nacional Sumaco Napo Galeras, trail from the Comunidad Mushullakta to Lluya Patcha (campamento #1) towards summit of Galeras, 1100–1200 m, 0°49'40"S, 77°33'49"W, 11 May 2011, *J.L. Clark et al. 12055* (ECUAMZ, SEL, US); • cantón Tena, Jatun Sacha Biological Station (Fundacion Jatun Sacha), forest between main cabin and Río Napo, 23 km east of Puerto Napo, 8 km east of Misahuallí, 485 m, 1°4'12.24"S, 77°37'46.46"W, *J.L. Clark 17485* (ECUAMZ, SEL, US); • La Joya de los Sachas, comunidad de Pompeya, lado sur del Río Napo, km 2 carretera de Maxus, 230 m, 0°28'S, 76°40'W, 26 Aug 1993, *M. Aulestia 356* (MO, QCNE, SEL); • 6 km south of Coca, towards Auca oil fields, Jul 1982, *L. Besse, H. Kennedy & R. Baker 1568* (SEL, US); • Coca, road to Las Aucas, 23 Jul 1977, *J.D. Boeke 2217* (NY, SEL); • Río Jivino, Limoncocha, 13 Mar 1968, *G. Harling, G. Storm & B. Strom 7631* (SEL); • Cañon de los Monos, ca. 12 km north of Coca (Puerto San Francisco de Orellana), 250 m, 4 Feb 1974, *G. Harling & L. Andersson 11725* (SEL); • Coca (Puerto San Francisco de Orellana), trail along Río Payamino, rastrojos, 250 m, 12 Feb 1974, *G. Harling & L. Andersson 11927* (SEL); • Río Sumino, tributary of the Río Napo, ca. 5 km northeast of Santa Rosa, 8 Sep 1968, *H. Lugo S. 204* (SEL, US); • Hongota at Río Mishuallí, ca 6 km east of Tena, 3 Apr 1969, *H. Lugo S. 1007* (SEL); • Santa Rosa at Río Napo, 27 Arp 1972, *H. Lugo S. 1950* (SEL); • Lago Agrio, 4 Feb 1973, *H. Lugo S. 3158* (SEL); • Coca (Puerto Francisco de Orellana), 17 Jan 1973, *H. Lugo S. 2791* (SEL); • environs of Limoncocha, 250 m, 16 Jun 1978, *M.T. Madison*, *T.C. Plowman & L. Besse 5331* (SEL); • Río Aguarico, environs of Dureno, downriver from Lago Agrio, 457 m, 1 Aug 1974, *T. Plowman, C. Sheviak & E.W. Davis 4035* (SEL, US); • along road from Napo to Puyo on way to Hacienda Dos Ríos, just outside Tena, above Mission Evangelica, near Río Tena, 3 Aug 1971, *H. Wiehler 71116* (SEL); • woods southeast of Tena, 4 Aug 1971, *H. Wiehler 71126* (SEL, US); • along road Hollin towards Loreto, 25 Apr 1983, *H. Wiehler et al. 93187* (SEL). • **Pastaza**: cantón Pastaza, parroquia Simón Bolívar, km 38 on the Puyo-Macas highway, near turn off towards Palora, 1000 m, 1°42'6"S, 77°50'36"W, 25 Jun 2003, *J.L. Clark & J. Katzenstein 8326* (QCNE, US); • cantón Mera, Sumak Kawsay In Situ reserve, ecological corridor Llanganates-Sangay, Río Anzu watershed, trail to parcela #2, 1211–1459 m, 1°24'25.91"S, 78°4'12.67"W, 9 Jan 2024, *J.L. Clark 17835* (QCA, SEL); • parroquia Veracruz, finca de Ursula Gelchsheimer, between Puyo and Macas, via a Taculín, entrada al Calvario, Río Bobonaza watershed, 609 m, 1°31'4.4"S, 77°50'21.03"W, 12 Jan 2024, *J.L. Clark, H.X. Garzón-Suárez 17882* (QCA, SEL); • cantón Mera, Río Anzu, 10–15 km north of Shell via dirt road, 1211 m, 1°24'39.75"S, 78°3'12.35"W, 14 Jan 2024, *J.L. Clark, L. Jost & H.X. Garzón-Suárez 17912* (QCA, SEL); • Puerto Sarayacu, at Río Bobonaza, 20 Mar 1971, *H. Lugo S. 1718* (SEL); • along road from Puyo to Macas, about 19 km outside of Puyo, near pond, 30 Apr 1979, *H. Wiehler et al. 79195* (SEL); • Río Anzu, road Mera-Río Anzu, 1000 m, 12 Feb 1990, *A. Hirtz 4586* (SEL). • **Sucumbíos**: Sacha Lodge, 3 km NW of the village Añangu, near the Napo river, 200 m, 0°30'S, 76°26'W, 8 Jun 1995, *J.L. Clark, L. Demattia & T. Miller 1060* (QCNE, US); • San Rafael, 25 Apr 1998, *H. Wiehler et al. 98150* (SEL). • **Tungurahua**: cantón Baños, parroquia Río Negro, patch of forest near Baños-Puyo road, western side of Río Topo, 1200 m, 1°24'16"S, 78°11'17"W, 27 Jun 2003, *J.L. Clark & J. Katzenstein 8402* (QCNE, US). • **Zamora-Chinchipe**: cantón Zamora, Jambo Bajo, eastern border of Podocarpus National Park, Fundación Maquipucuna, permanent plot 1, sector Nororiental, property of Sr. Jorge Acacho, 1100 m, 4°5'S, 78°55'W, 4 Nov 1996, *J.L. Clark, P. Conza, P. Walter & M. Zapata 3204* (MO, QCNE, US); • cantón Nangaritza, parroquia Zurmi, comunidad Centro Shaime (along Río Nangaritza), forest 2–4 km NW of Centro Shaime, forest on limestone outcrop, 1000 m, 4°18'6.3"S, 78°41'1.9"W, 14 Dec 2001, *J.L. Clark, A, Lucia, M. Terry & R. Chuinda 6492* (QCA, QCNE, SEL, UNA, US); • cantón Nangaritza, parroquia Zurmi, comunidad Centro Shaime (along Río Nangaritza), forest 2–4 km NW of Centro Shaime, 1000 m, 4°18'6.3"S, 78°41'1.9"W, 14 Dec 2001, *J.L. Clark, A, Lucia, M. Terry & R. Chuinda 6498* (QCA, QCNE, US); • cantón Zamora, buffer zone near the eastern border of Parque Nacional Podocarpus, near entrance of Copalinga Ecolodge, 955 m, 4°5'34"S, 78°57'38"W, 4 Jun 2007, *J.L. Clark et al. 9980* (ECUAMZ, US); • south of Yanzatza 850 m, 3 Feb 1987, *C. Luer, J. Luer & A. Hirtz 12612* (SEL). **Peru.** • **San Martín**: cantón Rioja, bosque Proteción Alto Mayo (BPAM), near Puente Aguas Verdes, confluence of Rios Aguas Verdes and Serranoyacu, km 397 on Highway 5N, carretera Fernando Belaunde Terry, 1170 m, 5°39'57"S, 77°44'54"W, 5 Jun 2010, *J.L. Clark et al. 11884* (SEL, US, USM); • cantón Rioja, Bosque de Proteción Alto Mayo (BPAM), near Puente Aguas Verdes, confluence of Rios Aguas Verdes and Serranoyacu, KM 397 on Highway 5N, carretera Fernando Belaunde Terry, 1170 m, 5°39'57"S, 77°44'54"W, 7 Jun 2010, *J.L. Clark et al. 11922* (SEL, US, USM); • cantón Rioja, distrito Pardo de Miguel, Aguas Verdes, 1234 m, 5°41'4.69"S, 77°39'28.25"W, 3 Jun 2024, *J.L. Clark, J. Flores, L. Valenquela & R. Rojas 18803* (HOXA).

#### Phenology.

Collected with flowers throughout the year. Collected with fruits in June.

#### Etymology.

The specific epithet honors Dr. Laura Vibiana Clavijo Romero, a preeminent botanist whose doctoral dissertation significantly advanced our understanding of *Drymonia* systematics. Clavijo’s extensive, collections-based research has greatly enriched our knowledge of Andean plant diversity, with a particular focus on Colombia and members of the flowering plant family Gesneriaceae. Currently serving as the director of the National Herbarium of Colombia (COL) and as faculty member at the Universidad Nacional de Colombia - Sede Bogotá, Dr. Clavijo has established herself as a leading authority on *Drymonia*. Her name has become synonymous with the study of this genus, and the specific epithet commemorates her invaluable contributions to the systematics and taxonomy of this group.

#### Distribution.

*Drymoniaclavijoiae* is the most widespread of the four species described here (Table 1). It is relatively common on the eastern slopes of the Andes, especially in Ecuador, southern Colombia, and northern Peru in premontane wet forests. It grows in the shade of forest edges and is often observed along roads in secondary and primary forests.

**Table 1.** General geographic distribution and comparison of morphological characters between *Drymoniacoccinea* and the four new species described here (*D.clavijoiae*, *D.katzensteiniae*, *D.rominieckiae*, and *D.silvaniae*).

	*Drymoniacoccinea* (Aubl.) Wiehler	*Drymoniaclavijoiae* J.L.Clark	*Drymoniakatzensteiniae* J.L.Clark	*Drymoniarominieckiae* J.L.Clark	*Drymoniasilvaniae* J.L.Clark
**Leaf blade length**	6–20 cm	8–22 cm	> 30 cm	10–26 cm	13–27 cm
**Corolla length**	3.5–5 cm	4–5 cm	< 2.5 cm	< 3.5 cm	to 2.8 cm
**Corolla color**	Uniformly yellow or yellow with dark spots on lower lobes	Uniformly yellow or yellow with dark spots on lower lobes	Uniformly orange (rarely orange suffused with yellow)	Usually yellow with red patches on dorsal corolla lobes (lobes rarely yellow with thin red horizontal striations)	White with prominent horizontal red striations throughout the corolla lobes and throat
**Inflorescence length**	< 10 cm	> 20 cm	5–7 cm	30 to 45+ cm	to 5 cm
**Inflorescence bracts**	< 5	5–15	5–10	5–15	5–10
**Inflorescence residue**	Not gelatinous	Gelatinous residue conspicuous	Not gelatinous	Not gelatinous	Not gelatinous
**Distribution**	Widespread in Amazonia & Guiana Shield (Bolivia, Brazil, French Guiana, Guyana, Suriname, and eastern Peru)	Eastern Andean slopes (Ecuador, southern Colombia, and northern Peru)	Eastern Andean slopes of Ecuador (Morona-Santiago, Pastaza, and Napo)	Eastern Andean slopes of southern Ecuador and northern Peru	Eastern Andean slopes of southern Ecuador (Zamora-Chinchipe)

#### Comments.

**Figure 1.**
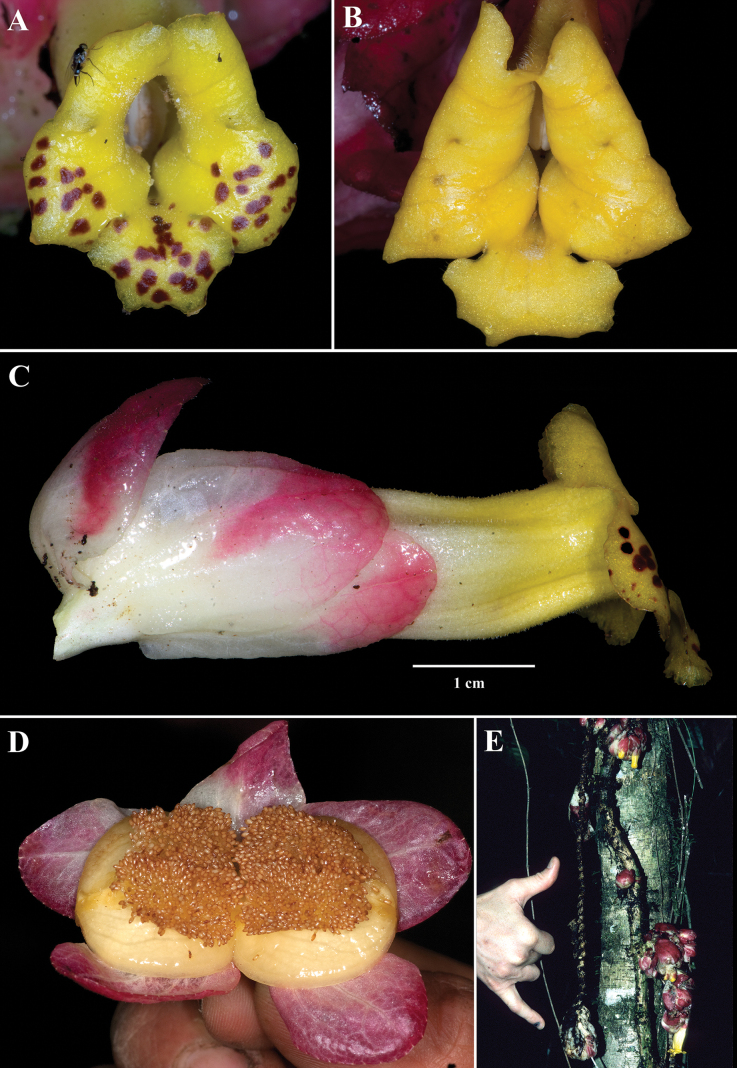
Field images of *Drymoniaclavijoiae* J.L.Clark **A, B** front views of corollas **C** lateral view of mature flower **D** fleshy bivalved capsule **E** elongate inflorescence at base of tree (**A** from *J.L. Clark et al. 11884***B** from *J.L. Clark 10233***C** from *J.L. Clark et al. 17912***D** from *J.L. Clark et al. 9980***E** from *J.L. Clark et al. 6492*). Photos by J.L. Clark.

**Figure 2.**
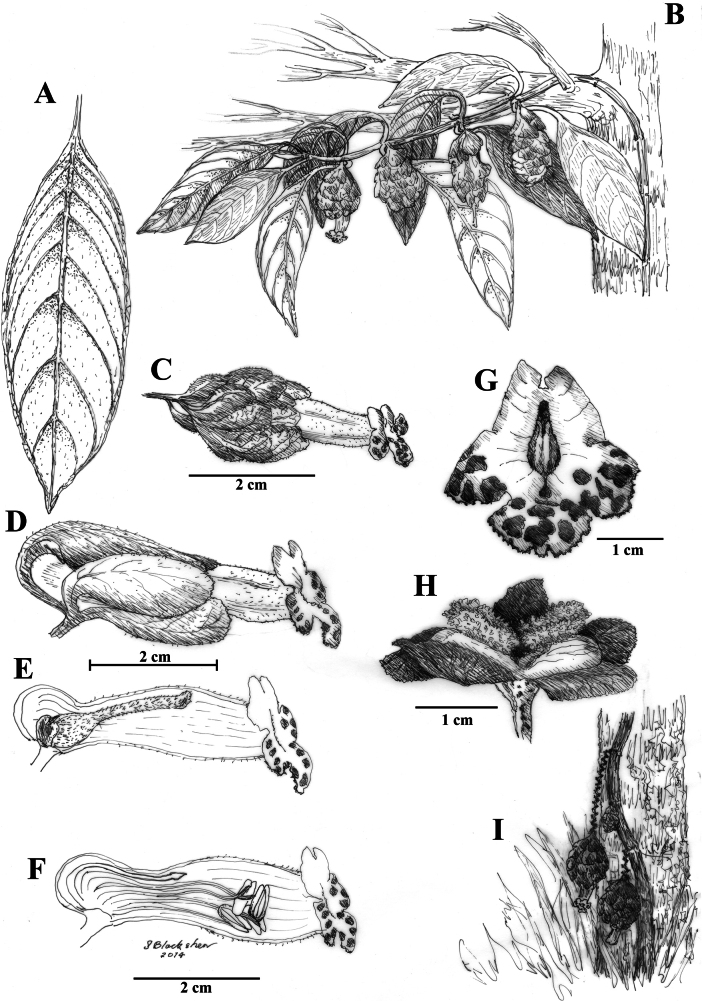
Illustration of *Drymoniaclavijoiae* J.L.Clark **A** adaxial leaf surface **B** habit featuring foliage and inflorescence **C** mature flower with inflorescence bracts **D** flower **E** lateral view of open flower with mature gynoecium **F** lateral view of open flower with mature androecium, including prominent staminode **G** front view of spotted corolla **H** fleshy bivalved capsule **I** elongate inflorescence featured at base of tree (**A** from *J.L. Clark et al. 9980***B** from *J.L. Clark et al. 9571***C** from *J.L. Clark et al. 9943***D, E, F** from *J.L. Clark 9473***G** from *J.L. Clark et al. 9943***H** from *J.L. Clark et al. 9980***I** from *J.L. Clark et al. 9943*). Illustration by Sue Blackshear.

Among the four species described, *Drymoniaclavijoiae* is the most widespread and most frequently collected. Its corolla tube is consistently tubular, yellow, and laterally compressed (Figs 1, 2). Most corolla lobes are yellow with brown spots (Fig. 1A), although some are uniformly yellow and spot-free (Fig. 1B). This species is characterized by clustered bracts, elongate inflorescence axes, and the presence of a gelatinous residue covering the flowers and inflorescences.

*Drymoniaclavijoiae* resembles *D.coccinea*, a species with an Amazonian distribution that includes the Amazon Basin and the Guiana Shield. The type locality for *D.coccinea* is French Guiana, based on a collection by Jean Baptiste Christophe Fusée Aublet (*Aublet s.n*., BM [barcode 000645637]) from Cayenne (Aublet 1775). Specimens from Amazonian regions (including the Guiana Shield) often exhibit compact inflorescences on the same stem region as the foliage. In contrast, *D.clavijoiae* typically has inflorescences and fruits near the ground (Fig. 1E), while its foliage is positioned in the subcanopy (Fig. 1B). Occasionally, both inflorescences and foliage are adjacent, but it is more common to find the flowers and fruits on a region of the scandent shoot near the forest floor and the leaves in the subcanopy. A tissue sample of *D.clavijoiae* (*J.L. Clark 6492*) was initially identified as *D.coccinea* and included in a phylogenetic analysis where it was strongly supported with other members of *Drymonia* that shared laterally compressed corollas (fig. 3 in Clark et al. 2015).

Documenting *Drymoniaclavijoiae* presents significant challenges due to the gelatinous residue or secretions covering its inflorescences. This sticky gelatinous exudate causes floral parts to adhere to newspaper during specimen preparation, often resulting in a hardened mass of compressed floral material and paper. Preparing the type specimen (*J.L. Clark et al. 9943*) required meticulous care, including multiple changes of newspaper throughout the drying process. Some specimens of this species were labeled with the herbarium epithet “umecta,” acknowledging the gelatinous residue and its medicinal use by natives of the region (Wiehler 1995).

The ecological significance of the gelatinous exudate in *D.clavijoiae* is unknown, but the presence of glandular structures and the production of secretory products has been the topic of several studies. Structural and functional roles of colleters or multicellular secretory structures were reviewed across 60 flowering plant families, where their presence was investigated for ecological roles in pollination, protection from pathogens and herbivores, or by reducing transpiration (Thomas 1991). It is noteworthy that Gesneriaceae was not listed among the 60 families by Thomas (1991) as having colleters.

Secretions on inflorescence bracts are well-known in members of the Bromeliaceae (Benzing 2000). A recent study by Ballego-Campos et al. (2023) investigated secretions, with an emphasis on trichomes in the floral bracts of 52 species of Tillandsioideae (Bromeliaceae). Other studies of secretions in Bromeliaceae have suggested that the gelatinous exudate acts as a sticky trap to protect against herbivory by insects, thus reducing damage to floral structures (Monteiro and Macedo 2014). It is likely that the gelatinous exudate in *D.clavijoiae* could play a similar ecological role in reducing herbivory, but further studies are needed.

### ﻿ Drymonia katzensteiniae

Taxon classificationPlantaeLamialesGesneriaceae

J.L.Clark sp. nov.

485620A3-0023-5629-8779-61C212E53A69

urn:lsid:ipni.org:names:77361652-1

Figs 3 , 4

#### Diagnosis.

Differs from *Drymoniacoccinea* by corollas that are less than 2.5 cm long (vs. corollas > 3.5 cm in *D.coccinea*), orange corolla tubes (vs. yellow corolla tubes in *D.coccinea*), and relatively large leaves that often exceed 30 cm in length.

#### Type.

Ecuador • Napo: parroquia Catundo, buffer zone of Parque Nacional Sumaco Napo Galeras, trail from the Comunidad Mushullakta towards crest of Galeras, 1100–1200 m, 0°49'40"S, 77°33'49"W, 25 Feb 2003, *J.L. Clark & N. Harris 7247* (holotype: SEL! [barcode SEL065816]; isotypes: MO, QCNE, US! [barcode US00818455]).

#### Description.

Elongate scandent subwoody nomadic lianas with leaves in the subcanopy (ca. 10 m above ground) and flowers produced near the forest floor along a leafless portion of the stem. ***Stems*** elongate and subwoody, terete in cross section, 3–6 mm in diameter. ***Leaves*** opposite, equal in a pair; petiole 2–8 cm long, green, terete in cross-section; blade broadly ovate to ovate, 13–27 × 5–10 cm, coriaceous, adaxially light green, abaxially green when alive and turning dark red when dry, apex acute to acuminate, base acute, sometimes slightly decurrent along the petiole, margin entire, 5–7 pairs of secondary veins, sparsely pubescent with single-celled trichomes abaxially, adaxially glabrous. ***Inflorescences*** of pair-flowered cymes that elongate from displaced bracteoles, produced along a leafless region of stem near ground, each often reaching 7 cm in length, with each inflorescence branch subtended by a pair of persistent bracts; bracts sparsely puberulent, oval and uniformly red, ca. 3.0 × 2.2 cm; each inflorescence with one open mature flower at a time. ***Flowers*** tubular and laterally compressed; pedicels 4–7 mm long. Calyx orange to orange suffused with red, glabrous, base of calyx with enations, lobes 5, nearly free, fused at the base for 2–4 mm, lobes overlapping, imbricate, and clasping corolla tube, broadly ovate, apex rounded, base broadly ovate, margins entire, ventral and lower lobes ca. 2.3 × 1.2 cm, the dorsal lobe slightly smaller, ca. 2.1 × 1.0 cm. Corolla tube zygomorphic, protandrous, oblique to perpendicular relative to calyx, ca. 3.2 cm long, gibbous at base, constricted laterally throughout, 7–10 mm wide, outside mostly glabrous at base and puberulent near apex, inside glabrous with minute glandular trichomes near apex, throat elliptic in cross section and constricted laterally, lobes 5, subequal, margins entire to erose, lobes reflexed, 5–6 × 7–8 mm, uniformly orange to orange suffused with red. ***Androecium*** of 4 didynamous stamens, included, filaments broad and flat, ca. 2.7 cm long, adnate to the corolla tube for 3 mm, white, glabrous; anthers oblong, sagittate, coherent by the lateral walls, initially dehiscent by basal pores that develop into longitudinal slits, 4–7 × 0.7–2.0 mm. ***Gynoecium*** with a single bilobed dorsal gland; ovary superior, 4.0–5.0 × 4.0–5.0 mm, cone-shaped, puberulent; style stout, included, 2.0 cm long; stigma stomatomorphic. ***Fruit*** a bivalved fleshy capsule, valves orange and reflexed when mature, each valve 1 × 1 cm. ***Seeds*** numerous, 0.8–10.0 × 0.4–0.5 mm, light brown, fusiform, ridged.

#### Additional specimens examined.

**Ecuador** • **Morona-Zantiago**: Río Namangoza, near Logrono, 550 m, 30 Dec 1976, *M.T. Madison & F.R. Coleman 2510* (SEL); • Cordillera de Cutucu, 25 km SE of Logrono, 1000 m, 30 Dec 1975, *M.T. Madison & F.R. Coleman 2525* (SEL); • Cordillera de Cutucu, 25 km SE of Logrono, 900 m, 17 Jan 1976, *M.T. Madison & F.R. Coleman 2617* (SEL-2 sheets); • Macas, along new road, west into the Andes, first 17 km westward, then ca. 12 km south on side road, 15 Apr 1988, *H. Wiehler et al. 8807* (SEL); • from Macas across Río Upano for about 15 km and then a 5 km hike by foot into the Cordillera de Cutucú, after 2.5 km across a river via steel basket on a cable, 16 Apr 1988, *H. Wiehler et al. 8836* (SEL); • above Patuca in the Cordillera de Cutucú, ca. 15 km from Patuca, in the area of the two waterfalls visible from the bridge, 19 Apr 1988, *H. Wiehler et al. 8898* (SEL). • **Napo**: cantón Tena, Jatun Sacha Biological Station (Fundacion Jatun Sacha), forest between main cabin and Río Napo, 23 km east of Puerto Napo, 8 km east of Misahuallí, 485 m, 1°4'12.24"S, 77°37'46.46"W, 10 Apr 2023, *J.L. Clark & J. Griefa 17596* (ECUAMZ, SEL, US); • Tena to Río Pano, 26 Apr 1993, *H. Wiehler et al. 93213* (SEL); Jatun Sacha, 28 Apr 1995, *H. Wiehler et al. 95133* (SEL). • **Pastaza**: cantón Mera, parroquia Shell, road to Río Anzu and beyond (south of the town Mera), trail heading north from road, 1350–1450 m, 1°23'15"S, 78°3'12"W, 6 May 2003, *J.L. Clark et al. 7795* (QCNE, SEL, UNA, US); • cantón Mera, town of Shell, small patch of forest on northern edge of Shell, 1100–1200 m, 1°29'14"S, 78°3'39"W, 7 May 2003, *J.L. Clark & M. Mailoux 7836* (MO, QCNE, SEL, US); • parroquia Veracruz, La Esperanza (Siguin), Finca Salina (de Hilda Perez), km 14 on the Puyo-Macas road, 800–1000 m, 1°32'40"S, 77°53'53"W, 24 Jun 2003, *J.L. Clark & J. Katzenstein 8307* (MO, QCNE, SEL, UNA, US); • parroquia Simon Bolivar, Bosque Protector Arutam (Fundación Arutam), km 47 on Puyo-Macas highway, 800–950 m, 1°46'53"S, 77°49'57"W, 10 Aug 2005 (QCNE, US); • parroquia Simon Bolivar, Bosque Protector Arutam (Fundación Arutam), km 47 on Puyo-Macas highway, 800-950 m, 1°46'53"S, 77°49'57"W, 10 Aug 2005, *J.L. Clark, D. Munez, S. Quinn & M. Katan 9160* (QCNE, SEL, US); • cantón Mera, Río Anzu Reserve (Fundacion EcoMinga), 10+ km along dirt road from Río Alpayacu, 1197 m, 1°24'9"S, 78°2'48"W, 2 Aug 2013, *J.L. Clark, J. Grammer, J. Martin & P. Taber 13653* (ECUAMZ, SEL, US); • cantón Pastaza, parroquia Veracruz, finca de Ursula Gelchsheimer, between Puyo and Macas, via a Taculín, entrada al Calvario, Río Bobonaza watershed, 609 m, 1°31'4.4"S, 77°50'21.03"W, 12 Jan 2024, *J.L. Clark & H.X. Garzón-Suárez 17868* (ECUAMZ, QCA, SEL, US); • 8 km from Puyo along road to Macas and Canelos, east along stream near distillery, 21 Apr 1986, *H. Wiehler et al. 86104* (SEL, US); • environs of Mera, hills east of town, 1188 m, 22 Nov 1974, *T. Plowman & E.W. Davis 4481* (SEL).

#### Phenology.

Collected with flowers in January, February, April, May, June, August, November, and December. Collected with fruits in June.

#### Etymology.

The specific epithet honors Jeanne Katzenstein, a horticulturist and life-long promoter for the taxonomic study of Gesneriaceae. Katzenstein has held several leadership roles in the Gesneriad Society, most notably serving as editor of the society’s journal for two decades (1992–2012). She was the Conference Organizer for the 2010 World Gesneriad Research Conference (WGRC 2010) held at Marie Selby Botanical Gardens. A tireless supporter of multiple generations of Gesneriaceae systematists, Katzenstein participated in 18 expeditions across Latin America with the late Hans Wiehler (1930–2003), who honored her contributions with an eponym, *Columneakatzensteiniae* (Wiehler) L.E. Skog & L.P. Kvist. Katzenstein co-directed four study-abroad programs focused on Gesneriaceae with John L. Clark and collaborated closely with Laurence E. Skog (Smithsonian Institution) to curate the Wiehler collections at Marie Selby Botanical Gardens. The species described here was collected by the author and Katzenstein during a collaborative 2003 field research expedition in Ecuador (*J.L. Clark & J. Katzenstein 8307*).

#### Distribution.

*Drymoniakatzensteiniae* is distributed in premontane wet forests along the eastern Andean slopes of Ecuador, particularly in the provinces of Morona-Santiago, Pastaza, and Napo (Table 1). Notably, it has not been recorded in the northern province of Orellana or the southern province of Zamora-Chinchipe. The species is typically found at elevations between 800 and 1200 meters, though some populations have been documented as low as 500 meters in Napo (e.g., *J.L. Clark et al. 17496*).

#### Comments.

**Figure 3.**
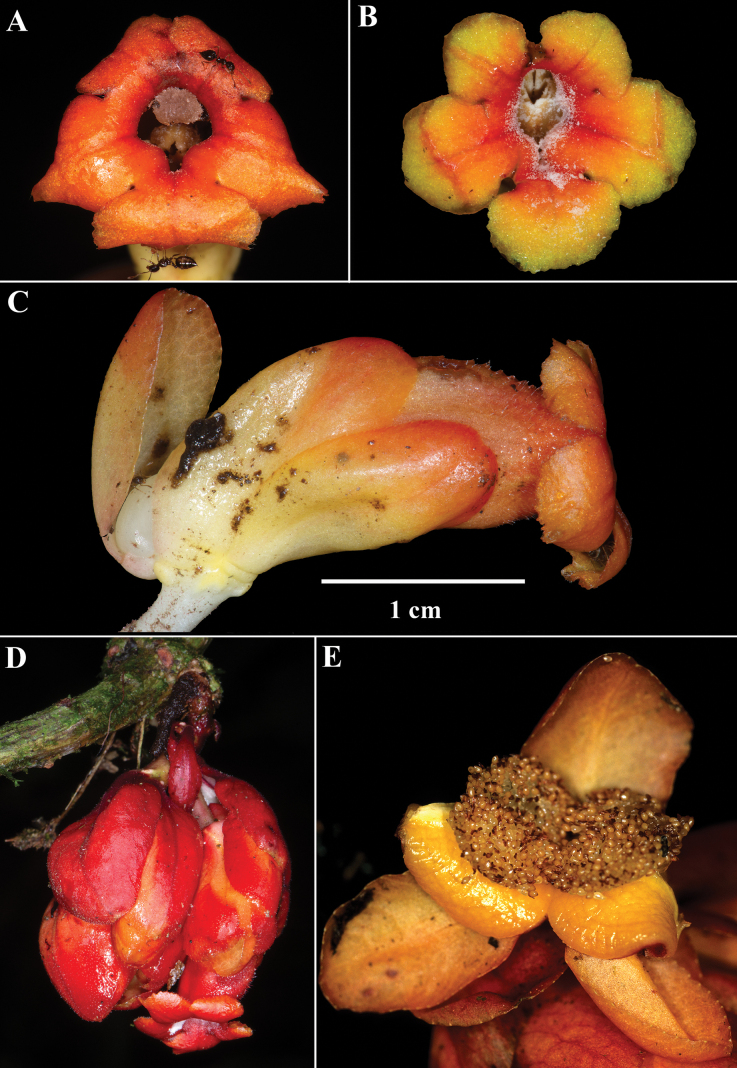
Field images of *Drymoniakatzensteiniae* J.L.Clark **A, B** front views of corollas **C** lateral view of flower **D** inflorescence **E** fleshy bivalved capsule (**A** from *J.L. Clark et al. 17496***B, C** from *J.L. Clark 13653***C** from *J.L. Clark et al. 17496***D** from *J.L. Clark et al. 17868***E** from *J.L. Clark 7795*). Photos by J.L. Clark.

Among the four species described here, *Drymoniakatzensteiniae* has the largest leaf blades (> 30 cm long) and the smallest flowers (corollas < 2.5 cm long). The bracts are reddish-orange, while the corolla tubes are orange, with the corolla itself ranging from orange to orangish-red (Fig. 3D). The corolla lobes vary from orange to red or red suffused with yellow (Fig. 3). Unlike *D.clavijoiae* (Fig. 1), the bracts of *D.katzensteiniae* are often moist but never gelatinous.

**Figure 4.**
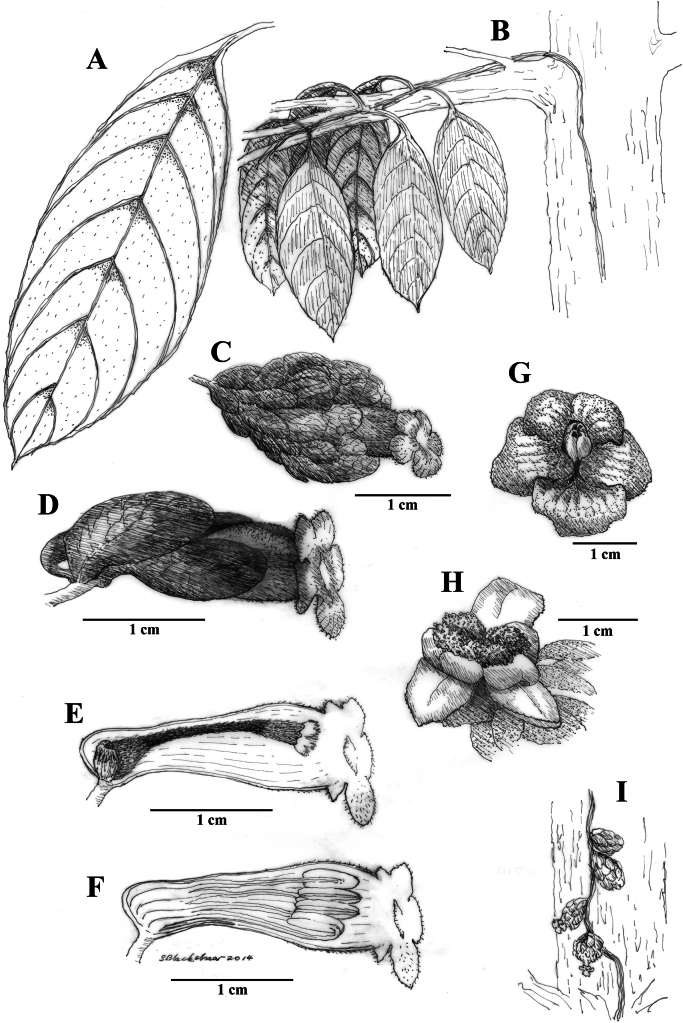
Illustration of *Drymoniakatzensteiniae* J.L.Clark **A** adaxial leaf surface **B** habit featuring foliage in subcanopy **C** mature flower with inflorescence bracts **D** flower **E** lateral view of flower with mature gynoecium **F** lateral view of open flower with mature androecium **G** front view of corolla **H** fleshy bivalved capsule **I** elongate inflorescence featured at base of tree (**A, B** from *J.L. Clark et al. 8307***C** from *J.L. Clark et al. 9160***D, E, F** from *J.L. Clark 13653***G, H** from *J.L. Clark 7795***I** from *J.L. Clark 13653*). Illustration by Sue Blackshear.

It is noteworthy that *Drymoniakatzensteiniae* is endemic to Ecuador and typically grows sympatrically with *D.clavijoiae*. Although *D.clavijoiae* is more widespread and abundant, occurrences of *D.katzensteiniae* often coincide with those of *D.clavijoiae* in the same localities. The overlapping distribution of these two species was a significant factor in the discovery and recognition of *D.katzensteiniae* as a distinct species.

Phylogenetic studies based on DNA sequence data have included samples of both *D.katzensteiniae* and *D.clavijoiae*, although these samples were initially determined as *D.coccinea*. There are no known samples of the currently recognized *D.coccinea* included in published phylogenetic studies or represented in the National Center for Biotechnology Information (NCBI) GenBank database. Tissue samples from *D.clavijoiae* (*J.L. Clark et al. 6492*) and from the type specimen of *D.katzensteiniae* (*J.L. Clark & N. Harris 7247*) were both recognized as members of *D.coccinea* in Clark et al. (2015). These two samples were resolved as sister-taxa in a phylogenetic analysis and strongly supported in a clade of *Drymonia* species that share laterally compressed corollas (see fig. 3 in Clark et al. 2015).

### ﻿ Drymonia rominieckiae

Taxon classificationPlantaeLamialesGesneriaceae

J.L.Clark sp. nov.

9736A180-0637-5936-8243-E8A81379A4B5

urn:lsid:ipni.org:names:77361653-1

Figs 5 , 6

#### Diagnosis.

Differs from *Drymoniacoccinea* by having elongate inflorescences (greater than 30 cm long) with numerous persistent bracts (usually many more than 10). In contrast, inflorescences in *D.coccinea* rarely exceed 10 cm and are typically composed of fewer than 5 bracts.

#### Type.

Ecuador • Napo: Zamora-Chinchipe, parroquia Zurmi, Comunidad Centro Shaime (along Río Nangaritza), forest 2–4 km NW of Centro Shaime, 1000 m, 4°18'6"S, 78°41'2"W, 13 Dec 2001, *J.L. Clark, K. Elmers, A. Lucia, M. Terry & M. Sharupe 6461* (holotype: US! [barcode US00818121]; isotypes: AAU, COL, F, K, MO, NY [barcode NY05154023], QCA, QCNE, SEL! [barcode SEL065815]).

#### Description.

Elongate scandent subwoody nomadic lianas with leaves in the subcanopy (ca. 10 m above ground) and flowers produced near the forest floor along a leafless portion of the stem. ***Stems*** elongate and subwoody, terete in cross section, 4–7 mm in diameter. ***Leaves*** opposite, equal in a pair; petiole 2–8 cm long, green, terete in cross-section; blade ovate to broadly ovate, 10–26 × 4–12 cm, coriaceous, adaxially and abaxially light green, apex acute to broadly acuminate, base acute, margin usually entire or rarely finely serrate, 5–8 pairs of secondary veins, sparsely pubescent with single-celled trichomes abaxially, adaxially glabrous. ***Inflorescences*** produced along a leafless region of stem near ground or in the subcanopy region with the foliage of pair-flowered cymes that elongate from displaced bracteoles, reaching up to 45 cm in length, each inflorescence branch subtended by a pair of persistent bracts; bracts puberulent, more so along veins, ovate and uniformly red, ca. 3 × 2.5 cm; each inflorescence producing one mature open flower near the apex at a time. Flowers tubular; pediels 6–10 mm long. Calyx uniformly red, puberulous, lobes 5, nearly free, fused at the base for 2–3 mm, lobes folded lengthwise with margins slightly recurved, not overlapping, but clasping corolla tube, lobes broadly ovate, apex rounded, base broadly ovate, margins serrate, ventral and lower lobes ca. 2.5 × 1.1 cm, the dorsal lobe slightly smaller, ca. 1.4 × 0.9 cm. Corolla tube zygomorphic, protandrous, oblique relative to calyx, ca. 3.5 cm long, gibbous at base, constricted laterally throughout, 5–9 mm wide, outside mostly glabrous at base and pilose near apex, inside glabrous with minute glandular trichomes near apex, throat elliptic in cross section and nearly constricted laterally, lobes 5, subequal, margins entire to erose, lobes reflexed, 4–6 × 5–7 mm, upper lobes with red patch, lower lobes uniformly yellow, rarely yellow with horizontal red striations. ***Androecium*** of 4 didynamous stamens, included, filaments broad and flat, ca. 3.0 cm long, adnate to the corolla tube for 3 mm, white, glabrous; anthers oblong, sagittate, coherent by the lateral walls, dehiscent initially by basal pores that develop into longitudinal slits, 4.0–6.0 × 0.5–1.9 mm. ***Gynoecium*** with a single bilobed dorsal gland; style stout, included, 2.0 cm long. ***Fruit*** a bivalved fleshy capsule, valves dark red and reflexed when mature, each valve 1.5 × 1.5 cm. ***Seeds*** numerous, 0.8–10.0 × 0.4–0.5 mm, light brown, fusiform, ridged.

#### Additional specimens examined.

**Ecuador. • Morona-Santiago**: Sucúa, along road to Los Tanques de Agua, ca. 12 km out of town, 18 Apr 1988, *H. Wiehler et al. 8879* (SEL, US). • **Napo**: road from Puyo to Canelos, km 31, 21 Jul 1982, *L. Besse et al. 1681* (SEL, US). • **Zamora-Chinchipe**: cantón Nangaritza, parroquia Nuevo Paraiso, Laberinto de las mil ilusiones, 1–2 km east of Río Numpatakayma (tributary of Río Nangaritza), 1000 m, 4°22'2"S, 78°39'39.6"W, 5 Mar 2018, *J.L. Clark 15583* (ECUAMZ, SEL); • Shaimi, Alto Nangaritza, 1000 m, 11 Jun 2004, *F.A. Werner 1311* (SEL, US). **Peru • Amazonas**: cantón Bagua, distrito Aramango, camino a Nueva Esperanza, Catarata Numparket, primary hiking trail between park guard station and crater, 1345 m, 5°28'34.53"S, 78°21'43.69"W, 6 Jun 2024, *J.L. Clark et al. 18958* (HOXA, SEL, US, USM); • Quebrada Huampami, 1 Nov 1972, *R. Kayap 11* (MO, SEL, US); • Bagua district, Aramango, trocha nueva Esperanza a la catarata bosque primario, 1650 m, 5°29'54"S, 78°20'00"W, 17 Dec 2001, *R. Vásquez, R. Rojas & L. Campos 27427* (MO, SEL, US). • **San Martín**: cantón Rioja, Bosque Proteción de Alto Mayo (BPAM), near Puente Aguas Verdes, confluence of Ríos Aguas Verdes and Serranoyacu, km 397 on Highway 5N, Carretera Fernando Belaunde Terry, 1170 m, 5°39'57"S, 77°44'54"W, 5 Jun 2010, *J.L. Clark et al. 11883* (COL, F, G, MO, NY, SEL, US, USM); • San Martín, cantón Rioja, Bosque Proteción de Alto Mayo (BPAM), near Puente Aguas Verdes, confluence of Rios Aguas Verdes and Serranoyacu, km 397 on Highway 5N, Carretera Fernando Belaunde Terry, 1170 m, 5°39'57"S, 77°44'54"W, 7 Jun 2010, *J.L. Clark et al. 11917* (USM, SEL, US).

#### Phenology.

Collected with flowers in March, April, June, July, November, and December. Collected with fruits in June.

#### Etymology.

The specific epithet honors Jennifer O. Rominiecki, President and Chief Executive Officer of Marie Selby Botanical Gardens. Rominiecki previously held prominent positions in New York City at The Metropolitan Opera, the Solomon R. Guggenheim Museum, and the New York Botanical Garden. Since assuming leadership of Selby Gardens in 2015, she has guided the institution through a period of remarkable growth, including the implementation of a three-phase Master Plan for rebuilding, which features the recently established Steinwachs Family Plant Research Center. At a time when many herbaria and collections-based research institutions across the USA and abroad have suffered setbacks, Selby Gardens has flourished under Rominiecki’s leadership, highlighted by the recent inauguration of a new herbarium that plays a vital role in advancing its mission.

#### Distribution.

*Drymoniarominieckiae* is distributed in premontane wet forests along the eastern Andean slopes of southern Ecuador and northern Peru at elevations between 800 and 1600 meters.

#### Comments.

**Figure 5.**
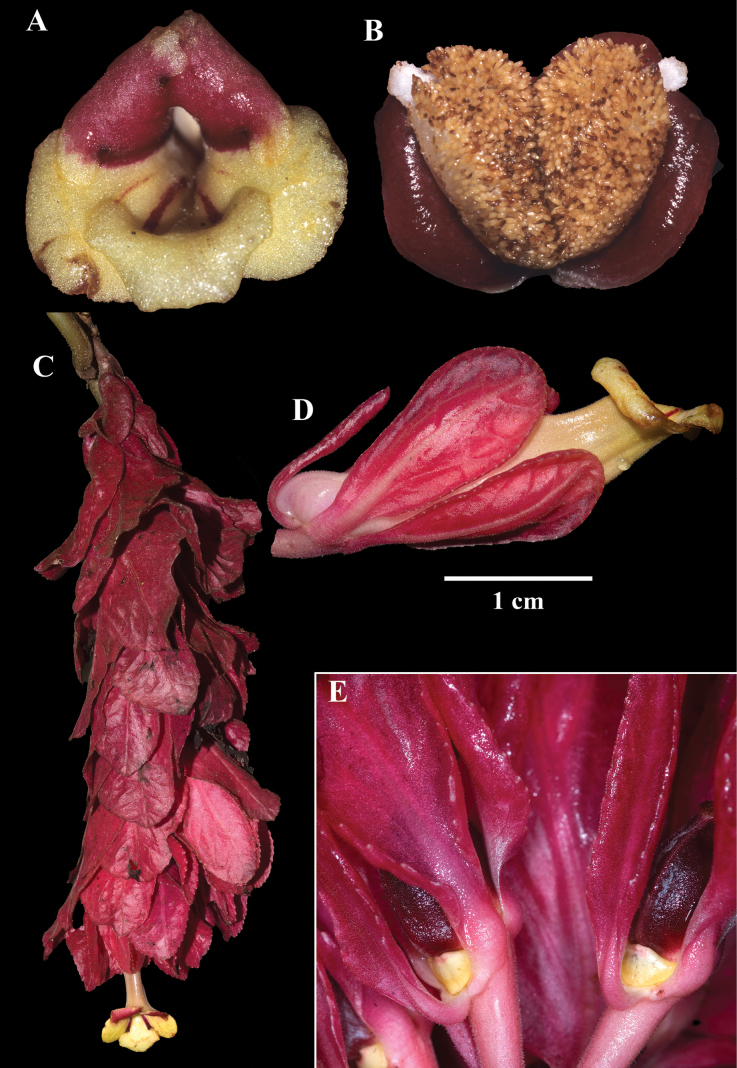
Field images of *Drymoniarominieckiae* J.L.Clark **A** front view of corolla **B** fleshy bivalved capsule **C** elongate inflorescence with persistent bracts **D** lateral view of flower **E** immature fruits with dorsal bilobed nectary gland (**A, B** from *J.L. Clark et al. 6461***C** from *J.L. Clark 15583***D** from *J.L. Clark et al. 18958***E** from *J.L. Clark et al. 11917*). Photos by J.L. Clark.

Among the four species described here, *Drymoniarominieckiae* has the longest inflorescences, one of which measured over 45 cm in length (*J.L. Clark et al. 11917*). This species tends to grow with lots of persistent showy red bracts, with some inflorescences bearing more than 40 persistent bracts (Fig. 5C). In contrast, the other three species often exhibit elongate inflorescences, but their bracts are not persistent and tend to fall off, exposing the zig-zag appearance of the inflorescence axis (Fig. 1E).

**Figure 6.**
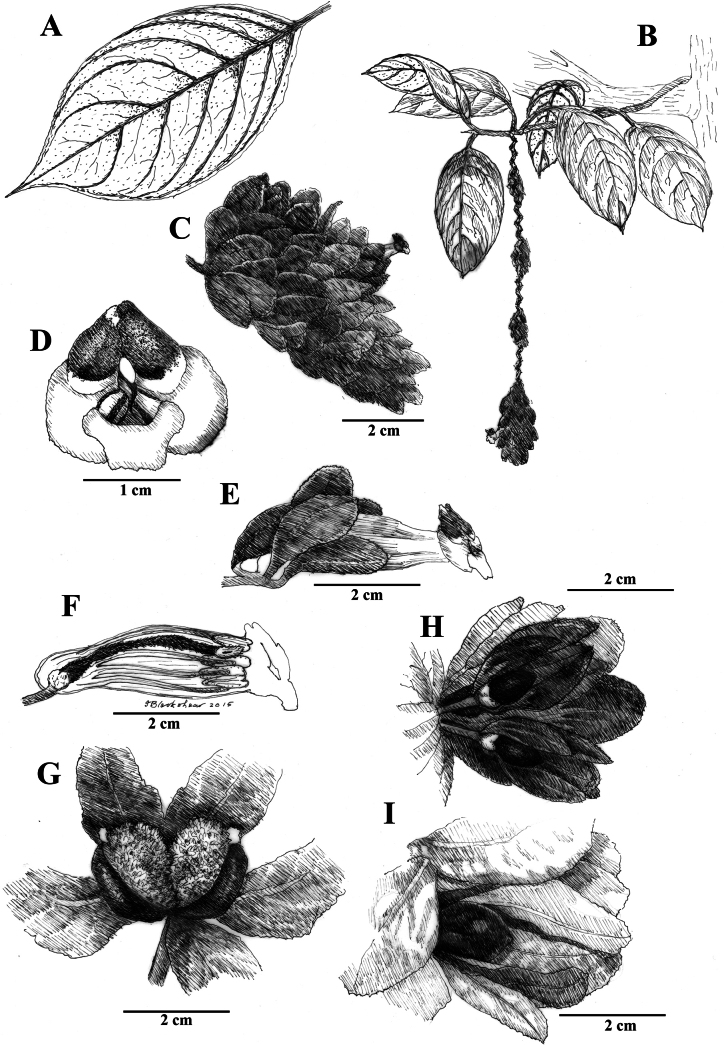
Illustration of *Drymoniarominieckiae* J.L.Clark **A** adaxial leaf surface **B** habit featuring foliage and inflorescence **C** mature inflorescence **D** front view of corolla **E** lateral view of flower **F** lateral view of open flower with mature androecium **G** mature fleshy bivalved capsule **H, I** immature cone-shaped fruits with nectary (**A, B** from *J.L. Clark et al. 11917***C, D** from *J.L. Clark et al. 6461***E, F** from *J.L. Clark et al. 11883***G, H, I** from *J.L. Clark et al. 11917*). Illustration by Sue Blackshear.

The corolla of *D.rominieckiae* is tubular, laterally compressed, and predominantly yellow (Fig. 5). The upper portion of the corolla limb and throat, especially the dorsal lobes, often with large red patches, while the lower portion, including the lateral and ventral lobes, remains yellow (Fig. 5A). Occasionally, the limb is yellow with thin, red horizontal striations (Fig. 5C) but is never uniformly yellow as seen in *D.clavijoiae* (Fig. 1B) or orange as in *D.katzensteiniae* (Fig. 2A, B).

**Figure 7.**
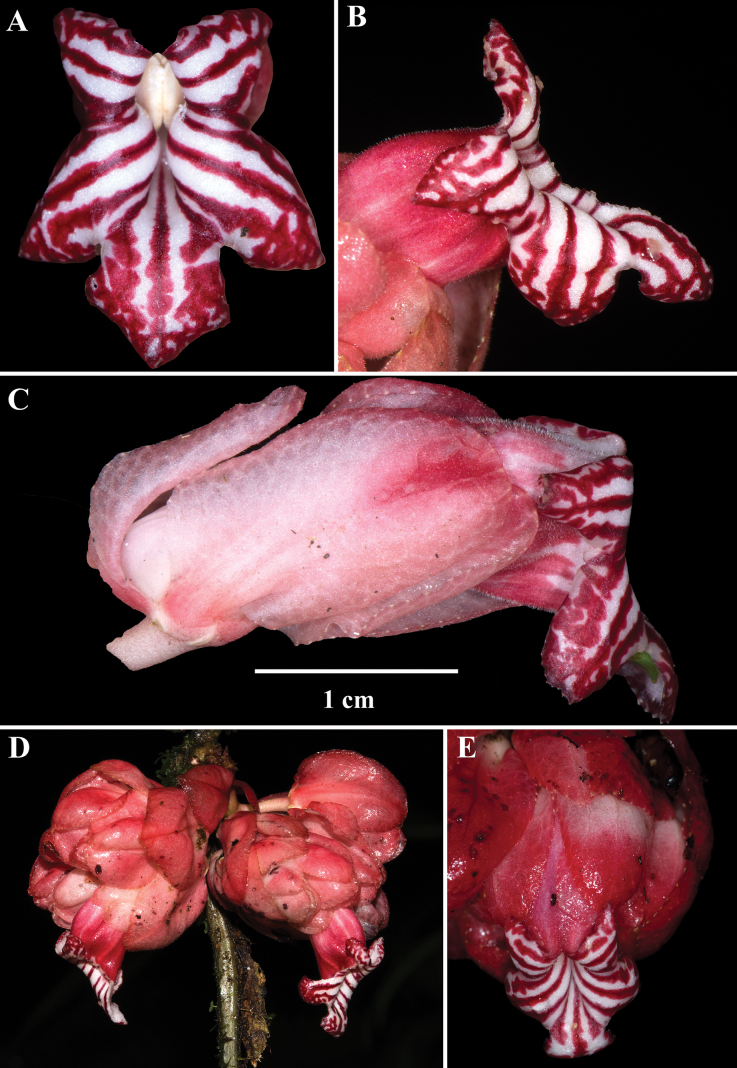
Field images of *Drymoniasilvaniae* J.L.Clark **A** front view of corolla **B** lateral view of corolla **C** lateral view of flower **D, E** inflorescence (**A, C** from *J.L. Clark et al. 15296***B, D** from *J.L. Clark et al. 16000***E** from *J.L. Clark et al. 10733*). Photos by J.L. Clark.

The calyx lobes of *D.rominieckiae* are distinctive, being obovate with a narrow base and a broad apex (Fig. 5D). This contrasts with the broadly oblong calyx lobes in *D.clavijoiae* (Fig. 2D), *D.katzensteiniae* (Fig. 3C), and *D.silvaniae* (Fig. 7C).

### ﻿ Drymonia silvaniae

Taxon classificationPlantaeLamialesGesneriaceae

J.L.Clark sp. nov.

1E2A0035-2547-557B-B40B-C4450A7DD8BE

urn:lsid:ipni.org:names:77361654-1

Figs 7 , 8

#### Diagnosis.

Differs from all other congeners by the longitudinal red striations contrasting with white on the inside of the corolla tube. Shares similar nomadic climbing habit with *D.coccinea* but differs by the shorter corolla (< 2.8 cm long vs corolla tube > 3.5 cm long) and relatively smaller leaves.

#### Type.

Ecuador • Napo: Zamora-Chinchipe, parroquia Zurimi, Cabañas Yankuam, Sendero Vino Tinto, trail on opposite side of road from Cabañas Yankuam, 800–1000 m, 4°15'54"S, 78°54'34"W, 12 May 2009, *J.L. Clark & University of Alabama in Ecuador Program Participants 10733* (holotype: SEL!; isotypes: ECUAMZ, US!).

#### Description.

Elongate scandent subwoody nomadic vines with leaves in the subcanopy (ca. 10 m above ground) and flowers produced near the forest floor along a leafless portion of the stem. ***Stems*** elongate and subwoody, terete in cross section, 3–6 mm in diameter. **Leaves** opposite, equal in a pair; petiole 2–6 cm long, green, terete in cross-section; blade broadly elliptic to ovate, 13–27 × 5–10 cm, coriaceous, adaxially light green, abaxially green when alive and turning dark red when dry, apex acute to acuminate, base acute, sometimes slightly decurrent along the petiole, margin entire, 5–7 pairs of secondary veins, sparsely pubescent with single-celled trichomes abaxially, adaxially glabrous. ***Inflorescences*** produced along a leafless region of stem near ground, of pair-flowered cymes that elongate from displaced bracteoles, often reaching 5 cm in length, each inflorescence branch subtended by a pair of persistent bracts; bracts uniformly puberulent, oval and uniformly red, ca. 1.5 × 1.5 cm.; each inflorescence with one mature open flower at a time; ***Flowers*** campanulate and laterally compressed; pedicels 5–7 mm long. Calyx white to white suffused with red, glabrous, base of calyx with prominent enations, lobes 5, lobes nearly free, fused at the base for 2–4 mm, overlapping, imbricate, but clasping corolla tube, lobes broadly ovate, apex rounded, base broadly ovate, margins entire, ventral and lower lobes ca. 1.8 × 1.2 cm, the dorsal lobe slightly smaller, ca. 1.5 × 1.0 cm. Corolla tube zygomorphic, protandrous, oblique to perpendicular relative to calyx, to 2.8 cm long, gibbous at base, constricted laterally throughout, 5–8 mm wide, outside mostly glabrous at base and puberulent near apex, inside glabrous with minute glandular trichomes near apex, throat elliptic in cross section and nearly constricted laterally, lobes 5, subequal, margins entire to erose, lobes reflexed, 5–6 × 5–6 mm, each lobe white with prominent horizonal red striations that extend into the corolla throat. ***Androecium*** of 4 didynamous stamens, included, filaments broad and flat, ca. 1.8 cm long, adnate to the corolla tube for 3 mm, white, glabrous; anthers oblong, sagittate, coherent by the lateral walls, dehiscence initially by basal pores that develop into longitudinal slits, 4.2–7.0 × 0.7–2.0 mm. ***Gynoecium*** with a single bilobed dorsal gland; ovary superior, 4.0–5.0 × 4.0–5.0 mm, cone-shaped, puberulent to velutinous; style stout, included, 1.4 cm long; stigma stomatomorphic. Fruit and seeds not observed.

#### Specimens examined.

**Ecuador** • **Zamora-Chinchipe**: cantón Nangaritza, Cordillera del Condor, trail from Cabañas Yankuam (west of Cabañas Yankuam) towards waterfall that is owned and operated by ATASMO (Asociación de Trabajadores Autónomos San Miguel de las Orquídeas), 912 m, 4°15'0.4"S, 78°39'36.4"W, 11 Mar 2017, *J.L. Clark, J.A. Mayr & D.A. Neill 15296* (ECUAMZ, MO, SEL, US); • same locality, 6 Mar 2019, *J.L. Clark & A. Wilcox 16000* (ECUAMZ, QCA, MO, NY, SEL, US).

#### Phenology.

Collected with flowers in March and May. Fruits not observed.

#### Etymology.

The specific epithet honors Silvana G. Nazzaro Clark, Head of School at Princeton Junior School (Princeton, NJ). The name *Silvana* is of Latin origin, derived from *silva*, meaning “forest” or “woodland,” and translates roughly to “woman of the forest” or “woodland maiden.” This name reflects a connection to nature, wilderness, and vitality—qualities Silvana has exemplified since childhood and carried into adulthood. She has accompanied and supported the author in a lifelong pursuit of studying biodiversity. Through her current and previous leadership roles, she has championed programs that nurture a new generation of lifelong learners. Her efforts have inspired a deep appreciation for the natural world, fostering curiosity and environmental stewardship in others.

#### Distribution.

*Drymoniasilvaniae* is endemic to the Ecuadorian province of Zamora-Chinchipe along the eastern Andean slopes in southern Ecuador at elevations between 800 and 1000 meters.

#### Comments.

**Figure 8.**
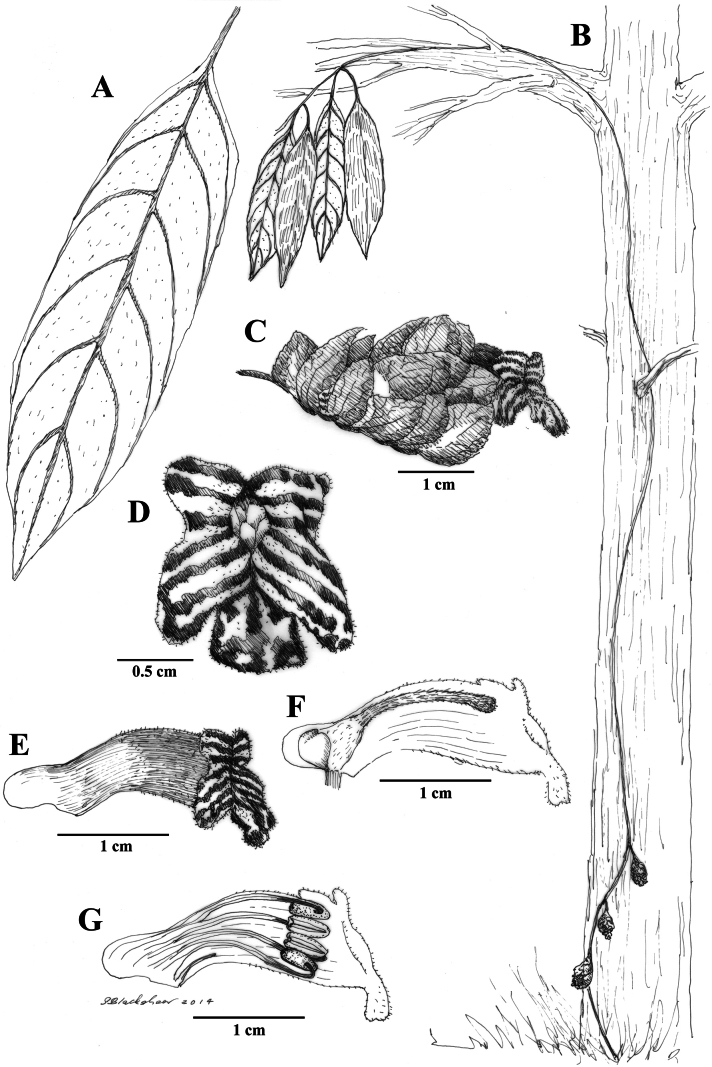
Illustration of *Drymoniasilvaniae* J.L.Clark **A** adaxial leaf surface **B** habit featuring foliage and inflorescence **C** inflorescence with mature flower **D** front view of corolla **E** lateral view of corolla **F** lateral view open flower featuring gynoecium **G** lateral view of open flower featuring androecium (**A–G** from *J.L. Clark et al. 10733*). Illustration by Sue Blackshear.

*Drymoniasilvaniae* displays a unique color pattern compared to any currently known species of Gesneriaceae. Its white, laterally compressed tubular corollas (Figs 7&8) are marked with elongate longitudinal striations, resembling the patterns found on candy canes often associated with the winter holiday season. The abaxial leaf surfaces dry a distinct dark red, sharply contrasting with the dark or light green surfaces observed in other species described here.

## Supplementary Material

XML Treatment for Drymonia clavijoiae

XML Treatment for Drymonia katzensteiniae

XML Treatment for Drymonia rominieckiae

XML Treatment for Drymonia silvaniae

## ﻿Additional information

### Author ORCIDs

John L. Clark https://orcid.org/0000-0002-1414-6380

### Data availability

All of the data that support the findings of this study are available in the main text.
